# The Logic of Pregnancy

**DOI:** 10.1093/jmp/jhad005

**Published:** 2023-04-20

**Authors:** Jonna Bornemark

**Affiliations:** Södertörn University, Huddinge, Sweden

**Keywords:** *Bracha Ettinger*, *law of identity*, *logic of life*, *Nicholas of Cusa*, *pregnancy*

## Abstract

This article takes its point of departure in Bracha Ettinger’s discussion on the “matrixial borderspace”: the structure of the experience of “the womb,” both from a “mother-pole” and a “fetus-pole”. Ettinger describes this borderspace as a place of differentiation-in-co-emergence, separation-in-jointness, and distance-in-proximity. The question this article poses is what kind of logic this experience is an expression of, as there seems to be a discrepancy in relation to the classical Aristotelian logic of identity. As an alternative to classical Aristotelian logic, Nicholas of Cusa’s logic of the non-aliud is explored as a paradigm more in line with Ettinger’s description of pregnancy specifically and more generally, to an understanding of life as a co-poietic emergence of structures of pactivity and permeability.

## I. INTRODUCTION

The experience of pregnancy is a peculiar one. It does not quite fit our ordinary experience of the world. In the ordinary experience we meet people face to face as they can come and go in and out of our field of perception. It is usually quite clear where one body ends and another one begins. If I am not in a psychosis, or similar condition, the self as well as the body would seem to hold together—at least as the ideal we are supposed to strive for.

In our everyday life, it is mostly unproblematic to accept that the chair is a chair and the table is a table. I am I, and you are you. The words fit the experience and are useful. But there are occasions when this no longer is the case; when every word seems to be too strong and say too much. Pregnancy is one of those occasions. Something is going on in pregnancy that does not fit our everyday language. Basic concepts, such as “one” and “two,” “self” and “other,” “me” and “you,” are no longer simple, but complicated and misleading.

But maybe the problem lies deeper than the choice of words? The kind of words that become problematic are about numerical identity, words necessary for counting: Are there one or two human beings in the pregnant body? There is an experience in pregnancy where both “one” and “two” says too much. In this article, I want to explore this gap between the logic of identity present in language and the phenomenon of pregnancy. And I want to explore whether there is another kind of logic that would fit this state of being more adequately.

To begin with, I will need to find a verbalization of the structure of the experience of pregnancy. Throughout the history of philosophy, explorations of the structure of pregnancy have not exactly been frequent. One reason is the male dominance in the discipline, another is that women who had the opportunity to write philosophy are often those that have not given birth, a typical example in the western tradition being medieval nuns. But in the last quarter of the 20th century, we have seen a growing interest in this subject, especially in the psychoanalytic tradition. If Julia Kristeva and Luce Irigaray might be called the founding mothers of this tradition, Iris Marion Young could be said to have brought it into phenomenology while researchers such as Joan Raphael-Leff elevated it into a topic of research in its own right.^1^ In the 21th century the philosophy of pregnancy has gained an increasing amount of attention.^2^ Throughout this literature the relation between the expectant mother and the fetus is thematized, together with the tricky question of to what extent, or in what way, this relationship should be understood as one between two separate entities.

In analytic philosophy this question has also been discussed, not least the ontological question of whether the pregnant body is one or two organisms. Barry Smith and Berit Brogaard have argued that 16 days after conception, when the process of gastrulation starts, the fetus is a tenant in a niche, that is, an individual substance on its own that lives within another individual and birth is just a move from one environment to another (Smith and Brogaard, 2003, 45–78). In a series of articles, Elselijn Kingma argues against this position.^3^ She claims that it does not hold as the placenta, umbilical cord, the chorionic and amniotic membranes, show that fetuses are not independent substances. The only solution might seem to be that the fetus becomes an individual substance first at birth. But that seems equally problematic, as that would imply a complete lack of continuity between the fetus before birth and the baby after birth. Kingma instead argues in favor of an understanding of the fetus as “lady parts”, a view entailing the idea that organisms can be part of other organisms, notwithstanding any metaphysical messiness and ambiguity this would imply. In connection to this, Kingma (2019) also points out that female philosophers, describing the phenomena of pregnancy, describe something very different from the image of being a container. In a sense, I want to continue Kingma’s line of arguing, but take it one step further by claiming that our dilemma here, in which we are forced into the position of choosing either to claim that the fetus and the bearer are two separate entities or to claim that they (up until the moment of birth) are exactly one and the same is the form of logic that is taken for granted. The conceptual messiness is only a problem from a certain logical perspective. I will therefore argue that there is another kind of logic that is more fruitful and more true to this phenomenon, in its ontological (and biological) sense as well as in its phenomenological and psychological sense. I will turn to another philosophical tradition than the one Kingma is addressing, and from a continental philosophical starting-point claim that carefully described, and reflected experience can be a source to understand the ontological question of “one or two” in pregnancy.

We will therefore turn to Bracha Ettinger, who wrote on this theme, especially during the 1990s and 2000s. She makes an elaborate analysis of the structure of intrauterine life and pregnancy, using psychoanalytical theory as a framework, but also picking up on thought elements from Emmanuel Levinas and Gilles Deleuze. While she does not directly discuss the discrepancy between pregnancy and the logic of identity, she nonetheless provides us with detailed material which we can analyze with such a question in mind. I will therefore use Ettinger’s discussion of “the matrixial borderspace” as an attempt to pick up some central elements of the structure of pregnancy.

After such an investigation, I will turn directly to the discussion of logic. First I will lay out the basic traits of Aristotelian logic, which is the basis for western formal logic, and then point out some discrepancies between it and the structure of pregnancy. Thereafter, I will turn to the 15th century philosopher Nicholas of Cusa to show how he develops another kind of logic that is not formal but rather interconnected with processes of becoming. Finally, I will discuss the relation between these two different logics and their relation to the matrixial borderspace (as Ettinger describes it), becoming, and the structure of life.

## II. INVESTIGATION

### Ettinger and the Matrixial Borderspace

Bracha L. Ettinger is an Israeli-French philosopher, psychoanalyst, and artist. In her *The Matrixial borderspace*, she explores the structure of pregnancy and of subjectivation in intrauterine life, claiming that life before birth is structured differently from life after birth. Ettinger criticizes Sigmund Freud and Jacques Lacan’s phallic psychoanalysis, with its focus on the self-formation and sacrifice of the womb for the sake of *oneness* (2006a, 55). She criticizes Julia Kristeva and Lacan when they claim that “the womb,” that is, the experience of intrauterine life and pregnancy, can only appear in psychosis and is the unthinkable par excellence (Ettinger, 2006a, 180). She describes a feminine sexual difference beyond the binary difference of the sexes, before and beside repression and castration (Ettinger, 2006a, 56). Lacan, Freud, and Kristeva thereby understand “the womb” as something uncanny and impossible to fully verbalize, whereas Ettinger claims that it can be investigated and calls it the matrixial stratum of subjectivation. To her “the womb” is not a lost paradise or a symbiosis beyond any conceptualization and understanding (Ettinger, 2006a, 70, 140), but, we could add, it needs a different kind of logic in order to do so. Ettinger claims that there is a missing piece in psychoanalysis, as the woman is either an attractive object of the father–son rivalry or a nursing object. She either belongs to copulating or nourishing—and there is a void between these two. What is missing is the pregnant and birth-giving mother who has been melted into obscurity by an ideological blindness (Ettinger, 2006a, 174). Here, there is an experience that is forgotten or silenced.

So, let’s focus on the experience of pregnancy, what does it look like? How is it structured?^4^ Ettinger uses the word “matrix” to describe a place where something develops or is molded. In this matrix there are not yet two: it is a place prior to the several and prior to both numerical and personal identity. What there are, are two (or three if there are fetus twins) *poles* (Ettinger, 2006a, 141). The mother is of course a person in a world with relations to other bodies that are already born, but this is not what she is in relation to the fetus. This state of being precedes motherhood and mother/child relationships (Ettinger, 2006a, 106–7). As we have all been fetuses we humans (and mammals) all have an experience of this state of being, but pregnant women have a particular form of double access: the womb as both past and present within (Ettinger, 2006a, 143). This is a state neither of total fusion nor total separation:

The matrixial is modeled on a certain conception of feminine/prebirth psychic intimate sharing, where the womb is conceived of as a shared psychic borderspace in which differentiation-in-co-emergence, separation-in-jointness, and distance-in-proximity are continuously reattuned by metamorphosis created by, and further creating—together with matrixial affects—relations without relating on the borders of appearing and disappearing, subject and object... (Ettinger, 2006a, 141)

We need to understand that the perspective here is the *inside* experience: The experience of the fetus and the experience of the mother-to-be in her relation to the fetus. But the words betray us since there is not yet “an experience of the fetus” or “an experience of the pregnant mother” as two separate strata. The experience is rather floating, not yet knowing where to draw the line between “myself” and “other”. There is not yet a constituted subject that can “have” these experiences, rather subjects arising through this very experience. It is in this sense that there are “not yet two.” Ettinger claims that this constitutes the structure of the experience of both the pregnancy itself and of the fetus, rather than a specific experience within the pregnancy or one of the fetus’ private experiences. To talk about “the womb” and “a matrixial borderspace” is an attempt to describe the place that these *not-yet two* have in common, the place in which they slowly grow forth as two. It is in this way that there is a co-joint co-emergence which nonetheless differentiates (Ettinger, 2006a, 72).

Ettinger claims that this includes a different kind of difference, what she understands as a feminine difference: relations without relating (2006a, 85). This “without relating” means that there are not yet two separate psyches that can relate to each other. There is rather a co-emerging I and not-I. There is a life characterized by the lack of a self as something separate from other selves. For the matrix creation is in “the im-pure zone of *neither* day nor night, of *both* light *and* darkness” (Ettinger, 2006a, 109). From such a starting point, stronger identities are growing forth. “Difference from a feminine angle diffracts; it is based on webbing of links and not on essence” (Ettinger, 2006a, 110). A difference based on “essence” would here mean numerical identities with fixed characteristics, identities which in a subsequent step overcome their separateness and can “meet” in a relation. This feminine differencing takes place in sensibility, but also in a birth of affection and cognition:

I and non-I are trembling in different ways along the same sensitive, affective and mental waves, sharing in different ways the same affective waves to create a feeling-knowledge of different aspects of a shared encounter-event. (Ettinger, 2006a, 120)

This does not mean that there are two individuals that have the same affections and cognitions. It is exactly this which is not at stage yet. The formation of affection and cognition are rather the formation of separate identities. But Ettinger claims that in the matrixial there is an affectionate room that individuals can be formed in.

Ettinger claims that compassion is an originary psychic manner of accessing the other. And, we should add, central in *wanted* pregnancies. But compassion here does not mean an individual that tunes in and sympathizes with another individual, it is rather a shared room of affection. This compassion is grounded in *com*-passion, a way to relate and be together without having the other as an object, and without an I. Com-passion is an openness and a sensitivity, an “I want this to be” that can be understood as an affirmation and a primary love. This com-passion is on the fetus-pole not yet connected to responsibility, but rather “directs a touching gaze to eternity and to the Cosmos” (Ettinger, 2006a, 128). Eternity should here not be understood as a harmonious whole, but rather as an experience where strict borders between the knower/feeler, and the known/felt is only about to take form, but not fixated.

As affection and sensibility are interconnected, the bearer understands the perception going on in the womb as a feel-knowing. In the shared space of pregnancy there is no subject–object division (as that would demand already clearly separated identities or essences), but an in-between-ness and uncognized co-presence. This “feel-knowing” is not structured in subject-object relations, but *of*, *by*, and *in* the other (Ettinger, 2006b, 100, 109).

The feel-knowing is also a capacity to respond, what Ettinger calls a co-response-ability where one part of the system responds to other parts, as a com-passionate affective, psychic, and mental resonance chamber. There are no distinct borders between oneself and the other in this foundational experience, but what she calls an almost otherness and a proximity rather than co-presence. On both the fetus-pole and the mother-side there is a *response-ability* before there is someone being responsible for someone else and there is com-passion before there is someone having compassion for someone else and a feel-knowing before cognition (Ettinger, 2006b, 111). Not to forget that from the mother-pole there also comes an already constituted subjectivity of responsibility and cognition, but in her pregnant state, in this pre-relation to the fetus, she is once again in a state of response-ability and feel-knowing.

To Ettinger it is also important that even though pregnancy has a special status here, those that are not pregnant are not cut off from this phenomenon. Both sexes have access to the matrixial via art and “compassionate joining-in-difference with others in transference relations” (Ettinger, 2006a, 143). We are all part of this matrixial web, as it is the place, or the experience, through which subjectivity is constituted. As such it lingers as a layer in all experience after pregnancy and birth. We carry it with us in our individual lives. Ettinger thus claims that this web is a trans-subjectivity that accompanies all subjectivity, even if its source is a pre-subjectivity. As such the matrixial conceptualizes the difference of what is joint but not the same, the uncognized in shared trans-subjectivity (Ettinger, 2006a, 183). She thus claims that individual subjectivity carries with it this origin also in adult subjectivity.

There is here a *differentiating-in-joining*, a becoming two through one and the same movement, in one and the same field of differentiating diffractions – a field it is impossible not to share (Ettinger, 2006a, 182). In this field, the poles that are initially more loosely organized, grow an increasingly harder skin, and borders towards the others arise, a process that continues also after birth. In this process psychic elements and threads more and more “belong” to increasingly fixed individuals. Knowledge grows as the separating movement takes place, knowledge about and within the matrix of I and non-I. Non-I is not already Other, but the co-emerging partial self and Other. In pregnancy, continuing after birth, there is a process of othering. Ettinger concludes that becoming-together thus precedes being-one (2006b, 47, 63, 72).

I will now turn to two different kinds of logic in order to analyze the logical structure of the experience of pregnancy as it is described by Ettinger. I will turn to Aristotle, as an example of the logic of being-one, and Nicholas of Cusa, as an example of the logic of becoming-together, and explore how their logics relate to the matrixial borderspace. These two thinkers have different understandings of the logical basic structure underpinning all philosophy, and they hold very different positions in the philosophical canon. Aristotle is extremely influential in western philosophy and logic, and to say that the commentator literature is vast is an understatement. Cusa on the other hand is less known and less influential. In order not to overflow space limits, I will here only pick up on some central themes in the logic of Aristotle and Cusa and discuss them in relation to the description of pregnancy above. This also means that I will stay within certain parameters of their oeuvres.

### The Law of Identity—The Logic of Aristotle

Let us begin by turning to Aristotle. In his *Metaphysics*, he formulates some basic tenets of western logic. He claims that without these elements, rational discourse would not be possible. These three interrelated axioms are:

The law of identity, which states that X = X:

First then this at least is obviously true, that the word “be” or “not be” has a definite meaning, so that not everything will be “so and not so”. Again, if “man” has one meaning, let this be “two-footed animal”; by having one meaning I understand this: —if “man” means “X”, then if A is a man “X” will be what “being a man” means for him. (Aristotle, 2009, Book IV, Part 4)

The law of non-contradiction, which states that “A is B” and “A is not B” cannot be the case at the same time:

It is impossible, then, that “being a man” should mean precisely not being a man, if “man” not only signifies something about one subject but also has one significance... And it will not be possible to be and not to be the same thing, except in virtue of an ambiguity, just as if one whom we call “man”, and others were to call “not-man”; but the point in question is not this, whether the same thing can at the same time be and not be a man in name, but whether it can be in fact. (Aristotle, 2009, Book IV, Part 4)

The law of excluded middle states that either A is true or not-A is true, as there are no other alternatives:

But on the other hand, there cannot be an intermediate between contradictories, but of one subject we must either affirm or deny any one predicate. This is clear, in the first place, if we define what the true and the false are. To say of what is that it is not, or of what is not that it is, is false, while to say of what is that it is, and of what is not that it is not, is true; so that he who says of anything that it is, or that it is not, will say either what is true or what is false. (Aristotle, 2009, Book IV, Part 7)

According to Aristotle, “the most certain principle of all is that regarding which it is impossible to be mistaken; for such a principle must be both the best known […], and non-hypothetical” (2009, Book 4, Part 3). They are “true of being qua being” (Aristotle, 2009, Book 4, Part 3). They are so self-evident that they do not really need any definitions. So how could anyone ever argue against them? Well, let us take a closer look at some of their elements and see if this is really the only way to think and make sense. I will pick up on their focus on definition and essence, on being and time, and on non-relationality. But there is yet another word that in this context cannot go unnoticed, and that is “man.” Aristotle belongs to a misogynistic tradition where the man is connected to reason (as can be seen in the quotations above). Without becoming essentialist, I still think we could note that this is a tradition of thinking where female experience, for example, pregnancy, is not taken into consideration. Women at large, and pregnant women especially, are the opposite of rationality.^5^ And this was not only the case in ancient Greece: throughout western history women, and especially pregnant women, have been called hysterical and in modern times it is “just hormones speaking.”^6^ The result is the same, she should not be listened to.

#### Definition and essence

Aristotle does not explicitly discuss the role of language in these laws. But it is obvious that language plays a central role. This becomes clear in the definition of truth given in the quotation on the law of excluded middle: “to *say* of what is...” (my italics). And in order “to say” words need to have singular, specific definitions:

It makes no difference even if one were to say a word has several meanings, if only they are limited in number; for to each definition there might be assigned a different word. For instance, we might say that “man” has not one meaning but several, one of which would have one definition, viz. “two-footed animal”, while there might be also several other definitions if only they were limited in number; for a peculiar name might be assigned to each of the definitions. If, however, they were not limited but one were to say that the word has an infinite number of meanings, obviously reasoning would be impossible; for not to have one meaning is to have no meaning, and if words have no meaning our reasoning with one another, and indeed with ourselves, has been annihilated; for it is impossible to think of anything if we do not think of one thing; but if this is possible, one name might be assigned to this thing. (Aristotle, 2009, Book IV, Part 4)

In rational discourse, each word needs to have one, and only one, specific meaning. And indeed, rational discourse greatly improves when we talk about the same thing and not about different things. But there is more to this attitude. To Aristotle this is not only about semantics, as the quote above may lead us to believe, but about *ontology* as well, or as he himself puts it, it is not about “whether the same thing can at the same time be and not be a man in name, but whether it can be in fact” (2009, Book IV, part 4). To have meaning means to relate to a precise and definite *essence*, that somehow exists “in fact”. “Definitions rest on the necessity of their meaning something” (Aristotle, 2009, Book IV, part 7). This “something” is beyond language, but is accessible and holds together in a unity. The laws of reason here say something not only about how we should organize language in the activity of reasoning, but also about the basic ontological structure of the world.

So, even if we agree on the need to have common definitions of central words in rational discourse, the idea that each word is connected to an essence thus need not apply to the structure of pregnancy. The field Ettinger describes is exactly not a place of fixed essences.

#### Time

Pregnancy is a becoming. It is a becoming that results in beings—beings that keep on becoming. With his logical foundations, Aristotle could be said to prioritize being over becoming (a statement which may not ring true of the entirety of his thinking). The basic structure of his argument is that one thing cannot *at the same time* be and not be. Time needs to be frozen when the law of identity takes its snapshot. Aristotle is here hunting for that which does not change and states with regard to his opponents that he must “persuade them that there is something whose nature is changeless” (2009, Book IV, part 5). It is central to his whole argument that the essences that the words belong to need to be changeless: Change may happen, but in each moment of time it is either the one or the other. This is a digital binary way to reason, long before digitals, as something either or is not. There are only two options, that is exactly the point of the law of excluded middle.

What *is*, is here beyond time, in frozen moments. This is also a foundational difference to Ettinger’s matrixial field where the movement of becoming is made the basis.

#### Non-relationality

Another underlying presupposition of Aristotle’s argument is that these essences are autonomous. X = X is a self-relation; it has nothing to do with the status of Y and Z. And relations only come after there are two or more separate *beings*. That is, identities exist firstly, we can only subsequently compare and explore differences and similarities, and build relations between them. There are essences, already separated, and as they are separated they can easily be counted. There is one and there are two.

These essences exist autonomously, disconnected from a wider context, and are closed in on themselves. The existence of closed-off essences is Aristotle’s vantage point: a point of departure that pregnancy or the matrixial field puts into question. In Ettinger’s description there are exactly no two separate entities relating to each other. There is a field of differences, a field with different poles—but lacking sharp borders.

#### Critics

The logic of identity is not as self-evident and uncontested as we might tend to think. In the Western tradition there has been a more or less constantly present subtext of criticism of it, explicit or implicit. Such a criticism has been present from pre-Socratic thinkers like Heraclitus,^7^ to Christian mystics such as John of the Cross^8^ and Theresa of Avila,^9^ to philosophers like Schelling,^10^ Derrida,^11^ and Deleuze.^12^ Nicholas of Cusa belongs to this group of thinkers.

### The Non-Aliud—The Logic of Cusa

Nicholas of Cusa lived in the 15th century and was active as a philosopher, cardinal, mathematician, and mystic in the apophatic tradition. He formulated a logic based on what he called the *non-aliud* and the coincidence of opposites. Cusa has a twofold relationship to Aristotle. On the one hand, he is part of a scholastic tradition where Aristotle provides the basic structure for all thinking with clear definitions and logic. On the other hand, he is a mystic in the apophatic tradition with a conviction that no words can describe God, and with a starting-point in movement rather than in fixed identities, and he therefore also develops a sharp critique of Aristotle. His way of resolving this tension is to argue that the logic of Aristotle should be integrated into a thinking that takes it beyond itself (Ziebart, 2013, 64–72).

In the following I will try to highlight how Cusa argues against some basic tenets of Aristotelian logic and how the logic of Cusa is more aligned with the structure of pregnancy. We should first however note that Cusa is writing in a theological tradition, aiming at relating to God rather than formulating a clear logic: the latter being a task we may need to help him with, through exegetic reconstruction.

#### The Law of Identity as Relational

In *De li non aliud*, a late text by Cusa, he discusses the law of identity and starts out with a question pertaining to knowledge:

NICHOLAS: I ask you, then, first of all, what is it that most of all gives us knowledge?

FERDINAND: Definition.

NICHOLAS: You answer correctly, for the definition is the constituting ground (oratio seu ratio). But on what basis is [definition] called definition? (1999, 1:3)

Here follows a long discussion which ends up explaining the importance of the expression “not other than” or in Latin *non-aliud* (Cusa, 1999, 1:3–5). This not-other could be understood as the equal sign in X = X. Non-aliud is what “=” stands for in this classic formula.

So, let us take a closer look at this concept. “Non-aliud” contains two negations, “not” and “other.” This points toward a kind of negative sorting mechanism: this is not that, nor that. It is a concept of identity, but its identity is foundationally relational, inherently built upon what it is not. What is not-other, must be the same. But this sameness is built upon what it is not. One central difference to Aristotle is consequently that Cusa by means of this expression emphasizes that the non-aliud includes all the things it is not, precisely by excluding them. Identity is thus not a pure affirmation of something in separation from everything else. On the contrary: identity is a relation to everything else as that which it is not.

A recurrent phrase in Ettinger is “non-I,” as the other that is not separated nor fused. The self is in this process the non-other, that is, that which is not other. In this movement of separating the one from the other, self and other, persons can grow forth. But in the matrixial, they are not as of yet two, they are in the midst of the non-aliud.

#### Difference-making power

Because other is other than something, it lacks that than which it is other. But because Not-other [non-aliud] is not other than anything, it does not lack anything, nor can anything exist outside of it. Hence, without Not-other [non-aliud] no thing can be spoken of or thought of (Cusa, 1999, 6:20).

The non-aliud is the power of separation which ties everything together as the power to separate is present in everything that is. The non-aliud is that which gives identity, cognition and thus undergirds all concepts. As such, Cusa understands it as a concept for “God”, “because God is not *other* than [any] other, He is Not-other” (1999, 6:21). We do not have to follow Cusa in his theological turn here, for our purpose it is enough to note that the non-aliud is central to the capacity to experience a differentiated world.

We can think about the perception of the fetus as a pure affirmation, but already here, in the recognition of something there is something else. The process of recognition is only possible through the possibility of *creating difference*, which Cusa understands as the power of the non-aliud. A certain melody sung close to the fetus is recognized as nothing but *this* melody: it is *not* heart beats or sounds of the intestine. This power is something the fetus is already capable of. In perception lies the capacity to experience something as exactly this—in difference to everything else. This capacity is also central to the creation of identity. Touching its own body is different from touching the surrounding uterus or the placenta, as it has a double character of being both touched and touching. Layer after layer of this experience (among other kinds of experiences) creates a difference between self and other. Separate individuals can thus emerge, both numerically and with personal identities.

This also means that when an identity grows forth, it does so in relation to others: to that which it is not but nonetheless always included in the self by exclusion. Non-I is not other as something unrelated, but rather something co-emerging. In pregnancy and infancy, the power of the non-aliud works its way through specific bodies. The experience of pregnancy is extreme in its embodied relation to the process of the non-aliud. The pregnant person experiences how something intimately and bodily intertwined with itself, step by step, becomes separated as an individual of its own and in relation to other bodies.

This is also connected to the movement of ideation, that is, of the origination of concepts. Cusa explains the relation between perceptions and ideas in the following way: “[f]or although intelligible things are not perceptible things, nevertheless they are not *other* than perceptible things. For example, coldness is not *other* than cold, as you stated” (Cusa, 1999, 14:53). “Just as the good is not *other* than the beautiful (as Dionysius says), so [it is] not [*other*] than any existing thing” (Cusa, 1999, 16:79).

The idea in its first stage is simply what lingers when the direct perception is gone. The sweet taste of apple in the amniotic fluid can linger in the memory of the fetus even after it is gone—making it possible to recognize it when it returns. This lingering of that which Cusa calls “whatnesses” (everything that has a content or meaning, has a “*quidditas*,” a “what”) is performed by the power of the non-aliud. The idea is not-other than the perception. Out of one thing (perception) comes another (idea) as neither identical, nor unrelated.

#### Movement

As a difference-making power, non-aliud is more than anything else a movement. It is the power of never staying in oneself: a continual drawing of temporary limits. This difference-making power could be understood as the power of life which continually erects a border around itself and becomes beings and individuals. The movement of the non-aliud continually takes form as the manifestation of *this* or *that*—it is not a power *beyond* concrete living beings, but *in* and *through* them. This logic is also present in Cusa’s earlier writings, from before his exploration of the concept of “non-aliud.” This logic has a structure of the one coming out of the other in a movement of simultaneous similarity and difference. So, let us follow how Cusa describes the emergence of knowledge according to such a logic. We could place it in the uterus, even if Cusa does not speak in such terms.

In the stream of perceptions that exist already in the uterus, there is thus the negativity of making differences, and, as we have seen, the capacity to “save” perceptions after they have left the presence of perception. Something we call memory or retention. This process opens up the possibility to experience what is not in perception but *could be* there. A capacity Cusa calls *imagination*. Imagination continues the movement of perception as it continues to affirm what is, but is separated from perception as it stands freer in relation to memory. It continues to build upon what *could be*, rather than that *which is*, thanks to the creation of a space for that which is not present, a space for negativity.

Imagination continues in the organization of ratio, which is also different from imagination, in the double movement of the non-aliud. Ratio continues the movement of imagination as its form does not come from perception: it thereby includes a creative element that is disentangled from the present. But ratio is different from imagination as it formulates opposites and abstractions. It is here that ideation takes place. The ratio defines its categories and counts, it formulates language and numbers. In this way it establishes a world “above” perception.

According to Cusa the intellect continues the movement of ratio and separates itself from it. They both relate to the world of concepts, abstractions, and order. But where ratio is focused on counting, intellect focuses on the basis of counting, the values and direction that picks up on some “whatnesses” and not on others. It sees and can criticize the whatnesses the ratio has picked up on and taken for granted. In the intellect Cusa claims that we can direct our gaze toward that which we apprehend as opposites: how warm and cold belong to temperature, and white and black belong to colors.

The capacity of the intellect is continued in and separated from not-knowing. Not-knowing continues the flexibility of the intellect and its gaze toward the horizons that surround human existence, but unlike the intellect, not-knowing does not create any new categories (Cusa, *On Surmises*, Part II, Ch. 16).

I will not discuss Cusa’s developmental idea of mental capacities any further, let us instead focus on the logic implied by it. This is a movement of the non-aliud. At every step of the way a new capacity can emerge in similarity and difference from the one before. Each capacity is thus not totally different from the one before. There is a gliding scale between, for example, memory and imagination, an analogical movement rather than binary digital differences.

#### Co-incidence of opposites

As we can see, in the intellect there is also a co-incidence of opposites. The intellect has the capacity to see what binds together that which we understand as opposites, for example, how color bind black and white together. Non-aliud more than anything else shows such a co-incidence of opposites. Cusa states that we now understand two things:

(1) that Not-other is presupposed and *known* in every cognition and (2) that what is known is not other than Not-other but is Not-other-qua-*un-known*, which shines forth knowably in what is known. (By comparison, in the visible colors of the rainbow, the clarity of perceptibly invisible sunlight shines forth visibly in various ways in various clouds.) (1999, 8:31, my italics)

Non-aliud, unrelated to everything else, cannot be understood. And yet it constitutes the power in every understanding. And even more, “for [Not- other] is not *other* than other but in other is the other” (Cusa, 1999, Prop. 18, 123). Non-aliud is thus not anything’s opposite, it is rather the otherness within everything. Being and identity is built upon the logic of differentiation-in-co-emergence, separation-in-jointness, and distance-in-proximity, to borrow Ettinger’s conceptualizations.

#### Language

**Fig. 1.**
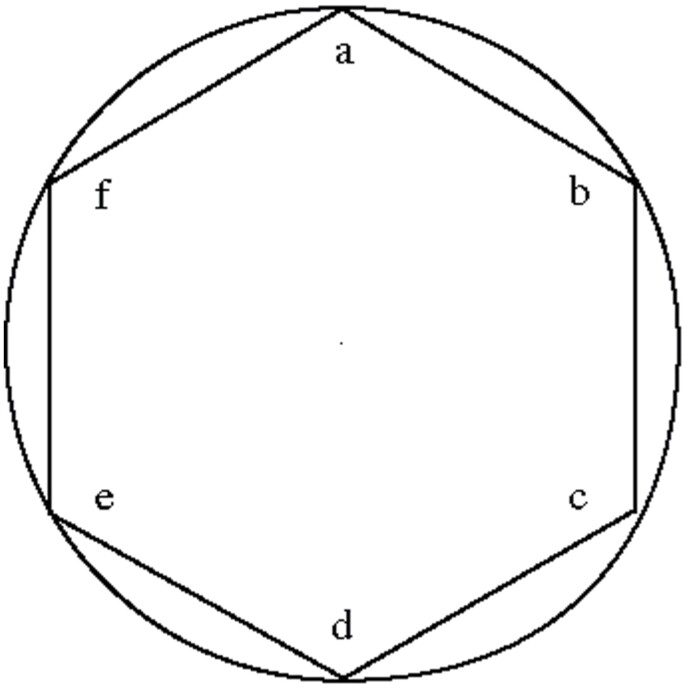
Drawing explaining the distinction between a round reality and polygonal concepts, presented by Cusa (2000) in chapter 7 of his book *On Surmises* from 1441-2.

But even if reality and experience is analog, Cusa claims that language needs to be digital. In language, humans seize “whatnesses” and provide them with a unitary form by separating them from other “whatnesses.” Cusa thus argues that our concepts always are polygons, that is, they have corners, whereas reality is round—a distinction he literally illustrates with a geometrical drawing (figure 1).

There is thus no perfect correspondence between language and reality. Language can never fully harbor reality, neither is language a pure construction. Instead, language continues the movement of picking up what is important, continues the movement of the non-aliud, and carves forth a “this.”

The ontological movement of non-aliud thus continues in language. In a logic of identity, language and being are autonomous, in the logic of non-aliud everything emerges in relation to each other, and also continues to change. Words as polygons continue a separating movement, and are thereby ill fit to capture a phenomenon like pregnancy. One strategy Ettinger uses in order to overcome this intrinsic limitation of language is to fully make use of hyphens. She uses hyphens in expressions like “differentiation-in-co-emergence” in order to point toward a field of intertwinement. But she also uses the hyphen to show that things we tend to understand as ready-made are really something that carry a history, for example, in concepts like “com-passion,” “feel-knowing,” and “response-ability.”

### The Logic of Life

By means of the logic of the non-aliud we can see how the logic of identity arises, how we constantly form identities and mistake them as essences on their own when we disregard their arisal. To use Cusa’s argument, from the ontological flux in the matrixial borderspace we pick up and form “whatnesses,” that is, essences. We build numerical and personal identities and once these are established and solidified we can use the logic of identity as formulated by Aristotle. The identity of logic is the continuation of this protection and develops further in language—understood as fixations of meaning. This logic has its place, debate would, for example, not be possible without it. This could be understood as part of life, as it continually searches to establish its borders. But at least human life seems to have a need of a forgetfulness of the becoming in order to protect its being (as that which it became).

The logic of the non-aliud is, as we have seen, closer to a logic of life. But neither can we fully stay in the logic of Cusa. Like Aristotle, he is also on the search for the immovable and infinite. He understands the non-aliud as something immovable in itself, as it creates the movement in everything else. We don’t have to follow him in this valuation of the immovable over the motile. The matrixial space of pregnancy speaks of something else, the power of a mutual dependency and incompleteness in itself. In a movement of the non-aliud, I would like to keep something (or quite a lot) from Cusa and add something new. Beyond and within the logic of the non-aliud, there is a logic of life. Let’s return to Ettinger and the structure of pregnancy and point out three characteristics inherent in such a logic of life.

#### Co-poiesis

Cusa stands in a tradition of a language of negativity, but in a logic of life it is not necessary to stay within these parameters. The movement of the non-aliud could here rather be understood as a process of co-poiesis. Co-poiesis is related to a tradition of autopoiesis, where, for example, life (but even other kinds of systems) arises through the development of its own inner structure (Ettinger, 2006a, 110, 159; see also Maturana and Varela, 1980). Such a creation is contrasted to a traditional causal understanding where the effect is separated and other than its causes. Life can here be understood as a “movement from within” and from its own premises—in difference to matter as pure *res extensa*, which is only moved by mechanical means. Co-poiesis emphasizes a becoming where processes of autopoiesis are not closed off and separated from one another. The movement of life does not belong to individuals, but rather to a diffracting movement through which individual life arises. This movement is not sub-ordinated the purpose of maintaining its own organismic identity. It is rather open-ended *at the risk* of its own identity (Ettinger, 2006a, 159). 

The diffracting movement of pregnancy also takes place in an intertwined perception. Intrauterine life is, for example, characterized by hearing rather than by sight. Hearing is not built upon distance and does not separate the one who hears from the sound in the same way as the seen is separated from the one who sees in vision. In sound there are frequencies, waves, resonance, and vibrations and in pregnancy an inside and outside vibrate together. The emerging two share and are shared by the same vibrating and resonating environment. There is a joint ontogenesis from which specificity and difference are derived (Ettinger, 2006a, 186). And there is a “co-emerging and co-fading in between life and non-life” (Ettinger, 2006a, 197). In a similar way the acoustic is not fully separated from other senses. Creating separation between two (or more) takes place as the difference-making activity of the non-aliud. In the matrixial sphere “it is the limit itself that is transformed by events in jointness, turning into a transgressive threshold” (Ettinger, 2006a, 179).

In co-poiesis, non-aliudity can thus be understood as a difference-making movement, creating a multitude and paving the way for identities to come and go in relation to each other.

#### Pactivity

As there are no autonomous beings in pregnancy, activity, and passivity must also be understood differently. In the logic of identity there is a tendency to think in causal relations where one part is active and acting, and the other passive and affected. This is not the case here, “[t]he matrix is a dynamic borderspace of active/passive co-emergence” (Ettinger, 2006a, 110).

In pregnancy there is an activity, but without control, since there is a “lodging in me without my self-control” (Ettinger, 2006a, 144). Giving birth is structured in this way. The labor is not something that can be controlled by the woman giving birth, but a power that comes from a place beyond her will. But neither is she passive, giving birth is very much an activity. The birth-giving woman is rather *pactive* (my concept, not Ettinger’s). That is, she has to take the waves of labor as they throw themselves over her, not only accept them, but make them her own, take them over, and either push or break them (by means of breathing). The logic of life is pactive. This is also the movement of the non-aliud as it partly continues and partly changes something: it continues a movement that comes from beyond, but can re-direct it as an active self is born.

Pactivity also speaks of contextuality. Being born is being born somewhere. We don’t choose our parents, country, language, and so on. As pactive beings we are formed by this heritage, but not stuck in it. We can take it over and give it another direction. Ettinger also notes that “imprints of non-I(s) are cross-scripted in *I*. Unknowingly, we participate in the traumatic events of the other, and art leads us to discover our part in events whose source is not ‘my’-self” (Ettinger, 2006a, 155). Affections are part of this pactive movement, and art is a way to deal with them.

By taking over what is given, active, and responsible, but not fixed, individuals can grow forth. But as non-aliud is a continual force, pactivity is continual and never completed once and for all.

#### Permeable bodies

Ettinger is, in her psychoanalytical vein, focused on a psychic, cognitive, affective sharing. But there is also an embodied sharing that lingers from our starting-point in shared embodiment: A living body only upholds its limits by letting these limits be permeable.^13^ A living body needs to breathe—letting air go through it. It needs to eat and defecate and urinate—letting nutrition go through it. And living bodies need to take place in each other in sexuality and pregnancy. We stay focused on clean borders, the skin as a fixed limit. But maybe living beings are rather characterized by their mucous membranes (for example, mouth, eyelids, nostrils, ureters, glans, vagina, anus, etc.). Especially those mucous membranes that are, or can be moist and with a passage between “inside” and “outside” of the body. These are sensitive areas where we can find the greatest pleasures, but where we are also the most vulnerable.

As permeable bodies, the non-aliud continues to work in us: keeping something and getting rid of something. A continual relation and non-relation to the manifold world around and within us.

## III. CONCLUSION

The experience of pregnancy is often an experience of a lack of words: or of words being too “strong” or “sharp”. Through a reading of Ettinger we could here nevertheless find a vocabulary and a way to relate to this experience, which appreciates its specific kind of ontology. By means of this verbalization, we can conclude that at least this part of life does not fit with a logic of identity. Within a logic of identity, we either need to understand the fetus as a separate being from the mother or as the same being as the mother. No third option would be available. In relation to Ettinger’s description of pregnancy, both these positions seem highly incorrect. Instead, following the lead of Nicholas of Cusa, we can note that a logic of life is more fitting. This is a logic of permeable, pactive bodies that arise in co-poiesis. By means of such a logic, we can understand not only pregnancy, but also more widely belonging, change, and mutual dependency. With the starting-point in a logic of life, we can also understand how beings become separated from each other and how whatnesses, through a negligence of becoming, can spring forth. It is this negligence that makes a logic of identity possible. Human systems such as law and rational arguments are built upon a logic of identity, and are important to human society. But we can nevertheless pose the question: what would happen if we gave more space and understanding to a logic of life? Maybe it would lead to a greater understanding of, for example, nature, bodies, and art?

## Footnotes

1 See Kristeva (1986), Irigaray (1985), Young (1984), Raphael-Leff (1993, 2015).

2 See, for example, Mullin (2005), Oliver (2010); LaChance Adams and Lundquist (2013), and Bornemark and Smith (2016).

3 See Kingma (2018, 2020a, 2020b).

4 A longer description of experiences in pregnancy, not only built upon Ettinger, can be found in Bornemark (2016).

5 The gendered structure of metaphysics is discussed, for example, in Lloyds (1984).

6 See, for example, Johannisson (1994).

7 Heraclitus’ thinking is characterized by a focus on becoming rather than on being, on the unity of opposites, and on the flux that connects everything. See fragments such as B91: “It is impossible to step in the same river twice, nor is it possible to touch a mortal substance twice in so far as its state is concerned. But, thanks to the swiftness and speed of change,] it scatters (things) and brings (them) together again, [(or, rather, it brings together and lets go neither again nor later, but simultaneously)] it forms and dissolves, and it approaches and departs.” (Heraclitus, N.d.)

8 In the Christian context it is especially in mystical traditions that an implicit critique of a logic of identity can be found. One such example is John of the Cross’ *Dark Night Of The Soul* (2000). The dark night takes place when the everyday logic dissolves and the difference between things is no longer clear.

9 Theresa of Avila’s *Interior Castle* (2004) can be interpreted as a journey away from a logic of identity into a different kind of logic and being. See, for example, Edith Stein’s (2006, 501ff) reading in the appendix of *Endliches und ewiges Sein – Versuch eines Aufstiegs zum Sinn des Seins*, where processes of objectification phase out the closer Theresa comes to God.

10 In German idealism it might be F.W.J. Schelling (1856–61, 138) that best personalizes the critique of the Aristotelian logic of identity the most. He criticizes the correspondence theory of truth as it presupposes an already fixed separation between object and subject and he also claims that the oneness of the self is not possible to reveal “if it is not an other in itself” (Schelling, 1856–61, 54).

11 Jacques Derrida’s critique of logocentrism as well as his deconstruction can easily be understood as a critique against a too simple logic of identity, see, for example, Derrida (1998).

12 Gilles Deleuze (2014) can equally be read in this vein. In *Difference and Repetition*, he argues that difference in western tradition has been reduced to identity, opposition, analogy, and resemblance, that is, a logic of identity. Instead he argues for an understanding of difference along lines of gradients, intensities, overlaps, etc (Deleuze, 2014, 50).

13 Permeability is of course a key theme also for other philosophers, see, for example, Merleau-Ponty (2002) and Butler (2006), but also Butler, Gambetti, and Sabsay (2016).
